# RETRACTION: Alternatively Expressed Transcripts Analysis of Non‐Small Cell Lung Cancer Cells under Different Hypoxic Microenvironment

**DOI:** 10.1155/jo/9836403

**Published:** 2025-12-19

**Authors:** Journal of Oncology

RETRACTION: S. Wang, H. Ma, H. Li, et al., “Alternatively Expressed Transcripts Analysis of Non‐Small Cell Lung Cancer Cells under Different Hypoxic Microenvironment,” *Journal of Oncology*, no. 2021 (2021), https://doi.org/10.1155/2021/5558304.

The above article, published online on 12 April 2021 in Wiley Online Library (https://wileyonlinelibrary.com), has been retracted by John Wiley & Sons Ltd.

The retraction has been agreed following an investigation undertaken by the publisher, which identified concerns related to Figure 1b of the manuscript. Specifically, the panels for the cells in group N share unexpected similarities after a vertical rotation.

The authors provided the raw data supporting the conclusions of the article; however, the data were not sufficient to address the concerns. As a result of this investigation, the results of the article are no longer considered reliable.

The authors disagree with this retraction and the wording of this notice.

